# Inhibitory Potential of Prodomain of *Plasmodium falciparum* Protease Serine Repeat Antigen 5 for Asexual Blood Stages of Parasite

**DOI:** 10.1371/journal.pone.0030452

**Published:** 2012-01-24

**Authors:** Asrar Alam, Virander S. Chauhan

**Affiliations:** Malaria Research Group, International Centre for Genetic Engineering and Biotechnology, New Delhi, India; Weill Cornell Medical College, United States of America

## Abstract

*Plasmodium falciparum* serine repeat antigen 5 (SERA5) is a target for both drug and vaccine intervention against malaria. SERA5 is secreted in the parasitophorous vacuole where it is proteolytically processed before schizont rupture. Among the processed products is a 50.8-kDa central domain of the protease, which possesses chymotrypsin-like activity and consists of a 28.9-kDa catalytic domain with a 21.9-kDa N-terminal prodomain, which remain attached together. Because SERA5 has been implicated in merozoite egress from host erythrocytes, the effect of the prodomain and a heptapeptide derived from its C-terminus spanning from D^560^ to F^566^ (DNSDNMF) on parasite growth was studied. When *E. coli*-expressed prodomain was incubated with parasite culture, a significant delay in transition from schizont to ring stages was observed up to nanomolar concentrations. The peptide, DNSDNMF also showed similar effects but at nearly 1000-fold higher concentrations. The peptide was also found to interact with the catalytic domain. These data demonstrate the crucial role of SERA5 prodomain for the egress process. Given the inhibitory potential of the prodomain for the parasite, we suggest that peptidomimetic inhibitors based on SERA5 prodomain sequences can be developed as future therapeutics against malaria.

## Introduction

Malaria caused by protozoan parasite *Plasmodium falciparum* is a global health burden causing enormous suffering and fatality in the tropical regions of the world [1]. Drug resistance and absence of a vaccine are the major factors leading to the spread of malaria. To control *P. falciparum* malaria, an effective vaccine and new drugs are urgently needed. One protein, which is important for both, is the highly expressed blood stage protein known as serine repeat antigen 5 (SERA5) [2]–[5]. SERA5 is a member of a large multigene family with 9 genes in *P. falciparum* transcribed most actively at trophozoite and schizont stages, of which eight (SERA1 to 8) are tandemly arranged on chromosome 2 and one (SERA9) on chromosome 9 [6]. All *P. falciparum* serine repeat antigens (SERAs) are transcribed most actively at trophozoite and schizont stages [7]–[9] at very different levels in a co-regulated manner [10]. Of these, SERA5 is most highly expressed with approximately 0.5–1.5% of the total mRNA in schizont stages [7] and released into the culture medium upon schizont rupture [11], [12].

To probe the essentiality of SERA genes for parasite blood stages Miller *et al.* (2003) and McCoubrie *et al.* (2007) were successful in deleting all the *P. falciparum* SERAs except SERA5 and SERA6 which were refractory to deletion [8], [13]. Expression of SERA5 mRNA increased two-fold in SERA4-null parasites suggesting a compensatory mechanism in these proteins [13]. Besides, blood stages SERAs have significant expression in other life cycle stages of the parasite indicating their roles in stages other than blood stages. Aly and Mutushewski (2005) have demonstrated that the disruption of the *Plasmodium berghei* ortholog of *P. falciparum* SERA8 led to the blockade of sporozoite release from oocysts [14].

SERA5 is secreted in the parasitophorous vacuole before egress and undergoes extensive proteolytic processing. It is processed into a 47-kDa N-terminal domain, a 50-kDa central protease domain, a 6-kDa fragment and an 18-kDa C-terminal fragment [2], [15], [16]. Recent reports have demonstrated that SERA5 and other SERA proteins are processed by PfSUB1, a parasite subtilisin-like protease [11], [17]. The central domains of all SERA proteins show ∼20% sequence identity with papain family cysteine proteases [18]. Phylogenetic analysis has revealed that SERA proteins can be grouped into two distinct groups, which have either serine or cysteine as the nucleophile [19]. In *P. falciparum*, six of the nine SERAs possess active site serine (SERA1 to 5 and SERA9) and three possess active site cysteine (SERAs 6 to 8). This non-conventional substitution of cysteine with a serine raised the question whether the SERAs with active site serine retained enzymatic function [8]. Hodder *et al.* (2003) showed that the 50-kDa central protease domain of *P. falciparum* SERA5 possessed chymotrypsin-like activity in *in vitro* assays [5].

Some of the SERA family members have been implicated in merozoite egress [11], [17]. Antibodies against the N-terminal 47-kDa region of the protein inhibited the transition of the parasite from schizont to ring stage but not from ring to schizont stage and also caused the agglutination of the released merozoites, suggesting the involvement of SERA5 in schizont rupture [20]. Sensitivity of egress and invasion processes to serine and cysteine protease inhibitors has attracted attention towards the role of proteases in these processes [11], [12], [14], [16]. Due to the presence of an active protease domain in SERA5 [5], its indispensability for parasite survival [8] and indirect evidences implicating SERA5 in merozoite egress from red blood cells [11], [17], the role of its proteolytic activity and the identity of its physiological substrates are tempting avenues to be explored.

The central protease domain of SERA5 (T^391^ to N^828^) is composed of a 187-residue prodomain (T^391^ to D^577^) which remains attached to the N-terminal of the 251-residue of enzyme domain (E^578^ to N^828^) after processing of SERA5 [5]. In case of zymogens, prodomains are known to have a variety of functions like prevention of premature activation of enzymes, proper folding of the protease domain, trafficking of the protease to its final destination and substrate recognition [21]–[23]; however, the physiological significance of the SERA5 prodomain remains unknown. In this study, we have attempted to explore the significance of SERA5 prodomain for the protein and role in merozoite egress.

## Results

### Cloning, expression and purification of SERA5 catalytic domain (SERA5 C) and SERA5 prodomain (SERA5 PD)

**Figure 1:**
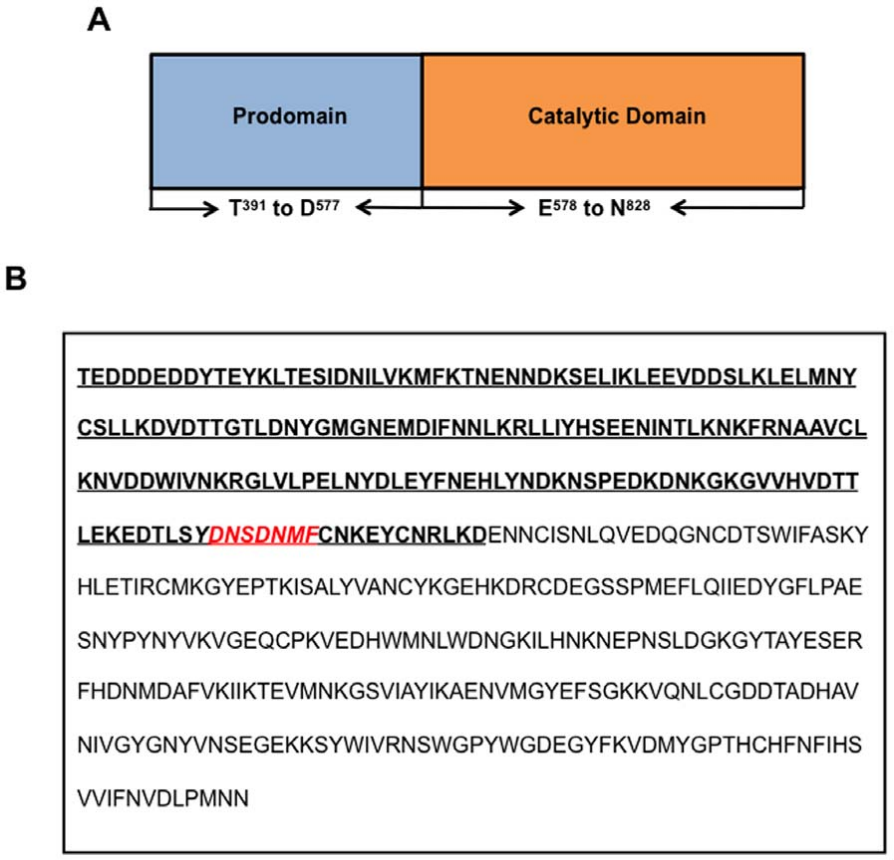
Architecture of SERA5 proenzyme. Panel A represents the general architecture of SERA5 proenzyme showing the position of prodomain and catalytic domain. Panel B shows the sequence of SERA5 proenzyme. Prodomain sequence is shown in bold and underlined. Catalytic domain sequence is shown in normal case letters. The inhibitory heptapeptide sequence is shown in red and italics.

Hodder *et al.* (2003) demonstrated weak proteolytic activity in the 50-kDa central enzyme domain of SERA5 (SERA 5PE). SERA 5PE consisted of a prodomain and a catalytic domain, whose boundaries were defined based on sequence alignment with known papain family cysteine proteases [5]. Figure 1A shows the schematic representation of SERA 5PE. Figure 1B shows the sequences corresponding to the prodomain and the catalytic domain. The complete prodomain polypeptide and a heptapeptide based on the C-terminal region of the prodomain were used for egress inhibition studies (Fig. 1A and 1B).

Due to the presence of a serine residue (Ser-596) in place of catalytic cysteine, weak protease activity and no information on the physiological substrates, protease activity of SERA5 is a matter of debate. We were interested in finding out whether the catalytic domain alone without the prodomain was proteolytically active. SERA5 C when expressed in *E. coli* BL21(DE3) cells with N-terminal hexahistidine tag accumulated mainly in insoluble fraction and was purified from inclusion bodies by nickel-nitrilotriacetic acid (Ni-NTA) chromatography (Fig. S1A). Refolded SERA5 C was found to be proteolytically inactive on fluorogenic substrates, Suc-Leu-Leu-Val-Tyr-AMC and Suc-Ala-Ala-Pro-Phe-AMC.

Hodder *et al.* (2003) have demonstrated that SERA 5PE underwent autolysis upon standing, resulting in a stable fragment SERA 5PEc, which leaves a heptapeptide (DNSDNMF) overhang at the N-terminal of the catalytic domain, which corresponds to the C-terminal of the prodomain [5]. We wanted to explore the role of the prodomain and the heptapeptide for the protein and merozoite egress. SERA5 PD, when expressed in *E. coli* accumulated mainly in the soluble fraction, which was purified by Ni-NTA chromatography followed by gel-permeation chromatography. Eluted protein peak (Fig. S1B) was collected, dialyzed against PBS, pH 7.4 and used for further studies.

### SERA5 prodomain inhibited schizont to ring stage transition

**Figure 2:**
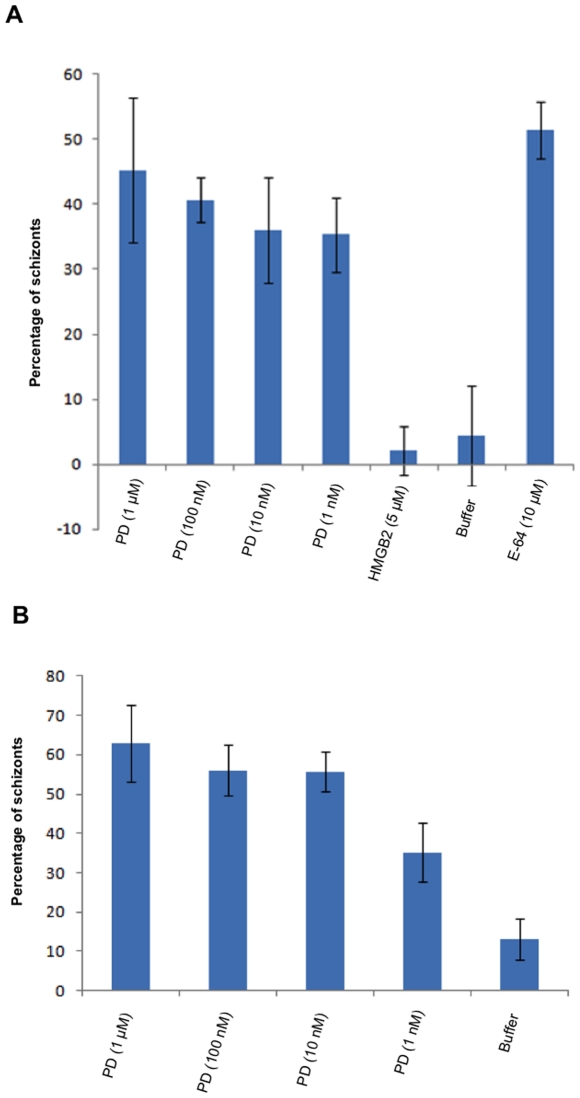
Inhibitory effect of SERA5 prodomain on parasite growth. Panel A: Schizonts were accumulated in a dose-dependent manner upon treatment of culture with SERA5 PD (referred as PD) at ring stage. Control *P. falciparum* protein recombinant HMGB2 had no effect on parasite growth. E-64 (10 µM) was taken as positive control, which caused significant accumulation of schizonts. Geimsa-stained smears of parasite culture were observed by light microscopy after 48 hours of treatment. Data is representative of mean of triplicate experiments. Error bars represent standard deviation of mean. Panel B: Schizonts were accumulated in a dose-dependent manner upon treatment of culture with SERA5 PD (referred as PD) at late trophozoite stage. E-64 (10 µM) was taken as positive control, which caused significant accumulation of schizonts. Geimsa-stained smears of parasite culture were observed by light microscopy after 24 hours of treatment. Data is representative of mean of triplicate experiments. Error bars represent standard deviation of mean.

To study the functional relevance of SERA5 PD for the enzyme domain, we monitored the effect of recombinant SERA5 PD on cultured asexual blood stages of *P. falciparum*. Effect of SERA5 PD on *P. falciparum* asexual blood stages was studied by addition of prodomain at ring stage parasites. We observed significant accumulation of shrunken unruptured schizonts in a dose-dependent manner after 48 hours post incubation up to nanomolar concentrations of SERA5 PD (Fig. 2A). A recombinant *P. falciparum* protein, high mobility group box protein 2 (HMGB2) prepared in our laboratory was used as a negative control and E-64, a broad-spectrum cysteine inhibitor was used as positive control in the above assay. Parasites treated with the control protein developed with normal morphology and were mostly present in ring stage after 48 hours (Fig. 2A). Significant populations of unruptured schizonts were seen in the E-64 treated culture whereas mostly ring stage parasites were seen in the buffer-treated culture (Fig. 2A).

To ascertain if SERA5 PD specifically blocked the transition from schizont to ring stages, recombinant protein along with E-64 was incubated with parasite culture at late trophozoite stage. A dose-dependent accumulation of schizonts was seen in culture treated with SERA5 PD up to nanomolar concentrations when compared with buffer-treated culture, which had mostly ring and trophozoite stage parasites at 24 hours post incubation. E-64-treated culture was found mostly in schizont stage (Fig. 2B). The final parasitemia at the time of scoring in both the above experiments was 4–5%.

### Localization of SERA PD inside infected RBCs

**Figure 3:**
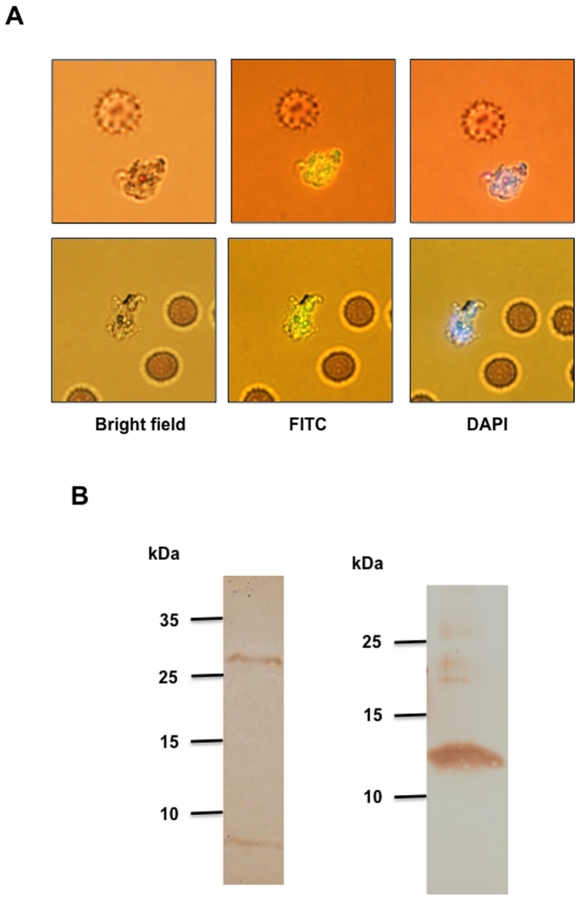
Localization of FITC-labeled SERA5 PD in schizont stage parasites after 48 hour of treatment. Panel A. Culture at ring stage was treated with 100 nM of FITC-labeled SERA5 PD and observed after 48 hours after counterstaining with DAPI. FITC fluorescence is seen in schizont stage parasites but absent in uninfected RBCs. Panel B. Signals corresponding to recombinant SERA5 PD and control protein HMGB2 (both with N-terminal hexahistidine tags) were detected in SERA5 PD- and HMGB2-treated parasite lysates respectively when probed with anti-hexahistidine antibody after 48 hours of incubation.

Upon incubation of ring stage parasites with fluorescein isothiocyanate (FITC)-labeled SERA5 PD, FITC-specific fluorescence was observed inside schizonts in live culture after 48 hours of incubation (Fig. 3A), suggesting that the recombinant protein crossed the erythrocyte and parasitophorous vacuolar membranes. Parasites were counterstained with DAPI to differentiate between the uninfected and infected RBCs. Hexahistidine-tagged recombinant SERA5 PD was detectable in the Western blot of SERA5 PD-treated parasite lysate after 48 hours of incubation, confirming that the recombinant protein was able to enter into the parasite (Fig. 3B).

### Interaction of the heptapeptide, DNSDNMF with SERA5 C

**Figure 4:**
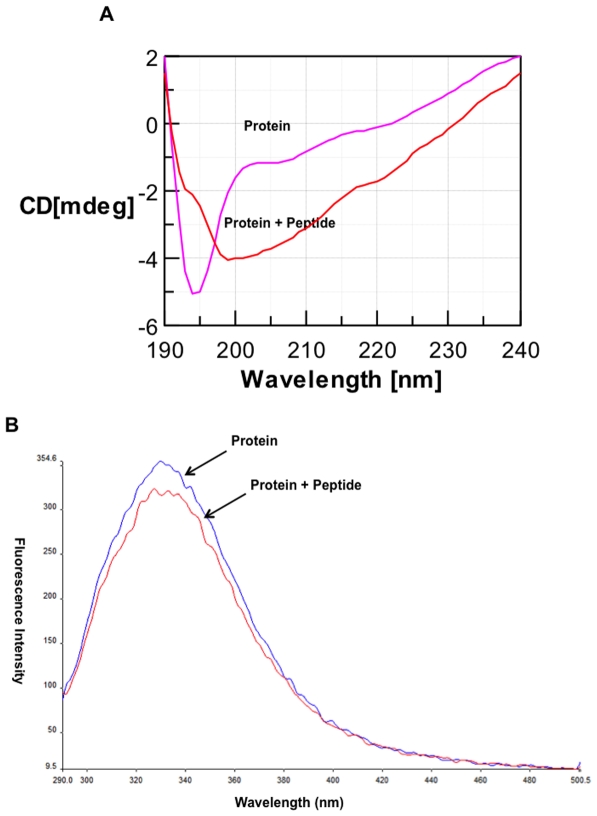
Interaction of heptapeptide, DNSDNMF with SERA5 catalytic domain. Panel A shows the interaction of DNSDNMF with SERA5 C by CD spectroscopy. The peptide induced significant conformational change when added with protein in 1∶1 molar ratio. Panel B shows the interaction of DNSDNMF with SERA5 C by spectrofluorimetry. The peptide induced quenching of the tryptophan fluorescence upon incubation with protein in 1∶1 molar ratio.

Interaction of synthetic peptide, DNSDNMF (Fig. S2) with SERA5 C was studied by CD spectroscopy and spectrofluorimetry. Upon addition of the peptide to SERA5 C (5 µM) solution in a molar ratio of 1∶1, significant conformational changes were seen in the CD spectrum of the protein, demonstrating the interaction of peptide with protein (Fig. 4A). Intrinsic tryptophan fluorescence emission spectrum of SERA5 C (1 µM) was recorded upon excitation at 280 nm and emission between 290 to 500 nm. Upon addition of peptide (1 µM), quenching in tryptophan fluorescence emission was seen, confirming the interaction between the peptide and the protein (Fig. 4B). These results demonstrated that the heptapeptide binds with the catalytic domain.

### Effect of the heptapeptide, DNSDNMF on transition from schizont to ring stage

**Figure 5:**
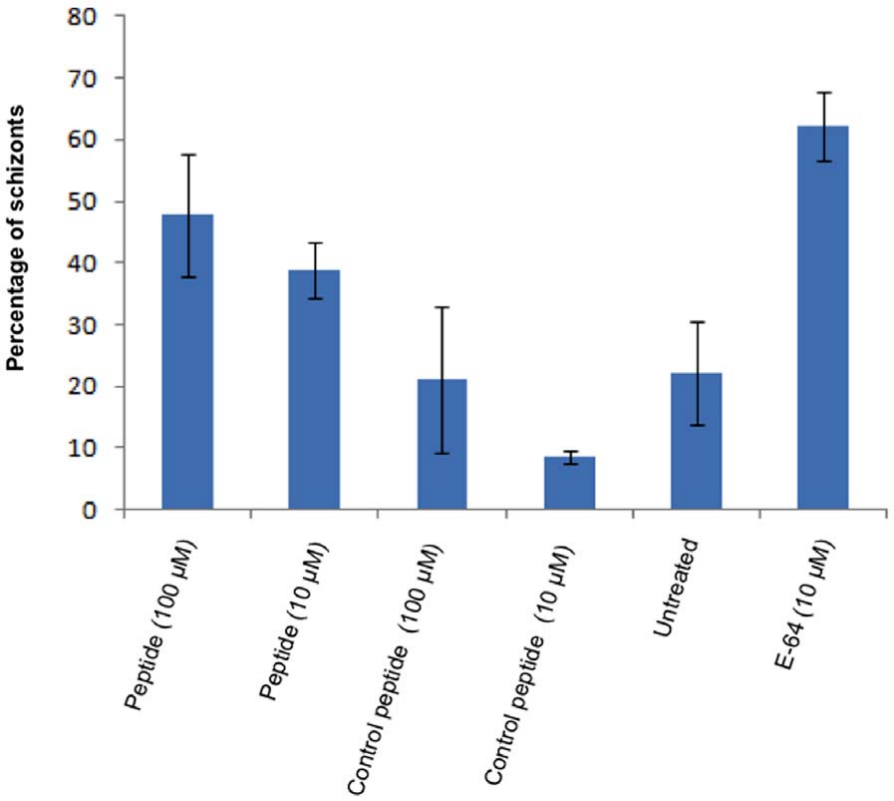
Inhibitory effect of heptapeptide, DNSDNMF on parasite growth. Culture was treated with peptide, DNSDNMF and control peptide, SIINFEKL at ring stage. Geimsa-stained smears were observed after 48 hours of treatment. Accumulation of schizonts was seen at 100 µM concentration of peptide, DNSDNMF. Control peptide, SIINFEKL did not have any significant effect on culture growth. Data is representative of mean of triplicate experiments. Error bars represent standard deviation of mean.

Incubation of DNSDNMF with parasite culture at the ring stages of the parasite resulted in accumulation of unruptured schizonts in a dose-dependent manner when observed after 48 hours of incubation (Figure 5). This effect was similar to incubation with the prodomain but occurred at ∼1000-fold higher concentrations. Culture treated with E-64 also showed accumulation of unruptured schizonts. To ascertain if the observed effects were peptide-specific, a control peptide, SIINFEKL (commercially procured from Techno Concept) was incubated with the culture at ring stage at 100 µM concentration. Parasites treated with the control peptide developed normally and were mainly found in ring stage. The final parasitemia at the time of scoring in the above experiment was 4–5% (Figure 5).

## Discussion

SERA5 is considered a potential drug target against malaria due to its indispensability for parasite survival [8] and its role in merozoite egress [11], [17]. Inside parasitophorous vacuole, SERA5 undergoes extensive proteolytic maturation by certain proteases. A serine protease belonging to subtilase clan, PfSUB1 processes SERA5 precursor into a 47 kDa N-terminal fragment and a central 56-kDa papain-like protease domain. The 56-kDa fragment in further processed by an unknown protease into a 50-kDa-fragment (SERA 5PE) [11] which possesses weak chymotrypsin like activity [5].

The present study was specifically focused on the significance of SERA5 prodomain (SERA5 PD) and catalytic domain (SERA5 C). Recombinant SERA5 C was found to be proteolytically inactive on fluorogenic substrates in *in vitro* assays described for SERA 5PE [5]. This observed loss of activity might be due to the absence of the prodomain, which may have chaperone like function and help in proper folding of the protease domain. Propeptides are known to have role in proper folding of their cognate proteases and in most cases they are cleaved upon maturation of the enzyme [22], [23]. In contrast, the prodomain of SERA5 remains attached to the protease domain and the whole PE domain is proteolytically active, hence the prodomain allows the entry of the substrates to the active site. Similar to the case of SERA 5PE, in human immunodeficiency virus-1 protease (HIV-1 PR), two β-strands of the prodomain called “flaps” fold on the active site of the enzyme and hence control the activity of the protease [24].

Although there are indirect evidences implicating SERA5 in merozoite egress [11], [17], the role of its protease activity is an area to be explored, particularly given the weak proteolytic activity of SERA 5PE and lack of information about the physiological substrates of the protease. Sequence alignment with other cathepsin L-like cysteine proteases revealed significant differences with classical proteases. Two other proteases, testin and silicatein, in which active site cysteine has been substituted with serine, do not possess proteolytic activity and perform different physiological functions [25]. Knockout studies of serine type SERAs in *Plasmodium berghei* have demonstrated that disruption of *P. berghei* SERA1 and 2 did not affect the development of parasites suggesting some auxiliary role of serine type SERAs (probably substrate recognition for the redundant cysteine type SERAs) [26]. Based on these findings, some regulatory roles for SERA5 other than proteolysis can be speculated. However, the exact role played by this enigmatic protein still remains a mystery.

Incubation of SERA5 prodomain (SERA5 PD) with cultured parasites at ring (6–8 hours) and mature trophozoite (32–36 hours) stages significantly delayed the transition of parasites from schizont to ring stage, which resulted in accumulation of unruptured schizonts in a dose-dependent manner. We suggest that there may be other functions for SERA5 prodomain besides regulating enzyme activity. Prodomains are known to be specific for their cognate proteases and carry out a variety of functions. Folding of the prodomain on enzyme active sites is a common phenomenon that keeps the enzyme in inactive form until certain physiologic conditions trigger the maturation of the enzyme. Maturation of the enzyme is accompanied by the proteolytic removal of the prodomain, which in many cases undergoes degradation by its cognate protease [27], [28]. Besides, inhibiting the active site, prodomains are also involved in numerous other processes including chaperone function, trafficking of the enzyme to its destination and substrate recognition [21], [28], [29]. Since, biological proteolytic events carried out by SERA5 are not known, the exact role played by the prodomain remains unclear. However, if the proenzyme form is considered active under physiological conditions, we may speculate that the prodomain may help in recognition and binding of the substrate and the addition of prodomain to culture may sequester the physiological substrate(s) from interacting with the enzyme. In a similar study, addition of recombinant prodomain of *Cryptosporidium parvum* subtilisin-like serine protease to parasite culture resulted in inhibition of infection of HCT-8 cells *in vitro* in a dose-dependent manner [30].

Earlier, Hodder *et al.* (2003) have shown that the major autolysis product of SERA5 proenzyme domain (5PEc) retains an 18-residue disulfide-bonded cyclic peptide at the C-terminal of the prodomain which might fold on the active site of the enzyme and further narrowed down it to the peptide sequence, DNSDNMF at the C-terminal of the loop, which was suggested to fold on the active site [5]. Interaction of this heptapeptide with the catalytic domain was confirmed by CD spectroscopy and fluorimetry, though the exact mechanism of this interaction is not clear. The peptide also delayed rupture of schizonts but at approximately 1000-fold higher concentrations of that with SERA5 PD.

Our findings are in agreement with the already suggested role of SERA5 in merozoite egress from RBCs [11], [17]. These effects of the prodomain and its derived peptide on parasite growth demonstrate the importance of the prodomain for the enzyme domain, but in absence of any information about the physiological significance of SERA5 proteolytic activity, it still remains an area to be investigated. Fairlie *et al.* (2008) have shown that a 14-residue long disulfide-bonded peptide selected by phage display screening targets the catalytic domain of SERA5 and delays the rupture of schizonts in a dose-dependent manner and hence demonstrated the potential of SERA5 as a drug target and its feasibility to be targeted by small molecules [31]. Hodder *et al.* (2009) solved the crystal structure of the catalytic domain; however, the peptide could not be co-crystallized with the catalytic domain [32].

Results of this study have also shown that prodomain of SERA5 is crucial for the protein and is inhibitory to merozoite egress. This finding also confirms the role SERA5 in merozoite egress and underscores its potential as a drug target. A heptapeptide derived from the C-terminal of SERA5 PD also had inhibitory effect on merozoite egress suggesting that peptidomimetic inhibitors based on the SERA5 prodomain sequences can be developed as future antimalarials.

## Materials and Methods

### Preparation of recombinant SERA5 catalytic domain (SERA5 C) and SERA5 prodomain (SERA5 PD)

SERA5 (PlasmoDB ID: PFB0340c) catalytic domain encoding region (*sera5c*) and prodomain encoding region (*sera5pd*) were PCR amplified from cDNA of asynchronous *P. falciparum* 3D7 parasites by using the primer pair: forward (S5CFw) 5′-GGCGCGGATCCGAAAATAATTGTATATCTA ATCTTCAAGTTG-3′ and reverse (S5CRv) 5′-GGCCGCTCGAGCTAATTATTCATAGGTAAATCAACATTGAATATAAC-3′ and forward (S5PDFw) 5′-GGCGCGGATCCACAGAAGATGATGATGAAGATGATTATACTG-3′ and reverse (S5PDRv), 5′-GGCCGCTCGAGCTAATCTTTTAATCTGTTACAATATTCTTTATTACA-3′ respectively. PCR products were cloned into pGEM-T Easy vector (Promega) and sequenced by dideoxy chain termination reaction. After sequencing *sera5c* and *sera5pd* were subcloned into pProExHTb vector (Invitrogen) between BamHI and XhoI sites with N-terminal hexahistidine tag. SERA5 C and SERA5 PD were expressed in *E.coli* BL21(DE3) cells. Expression was induced with isopropyl β-D-thiogalactopyranoside for 4 hours at 37°C and cells were harvested by centrifugation.

For purification of SERA5 C, cell pellets were suspended in lysis buffer (10 mM Tris-HCl, 10 mM EDTA, 100 mM NaCl, 5 mM benzamidine-HCl, 10 mM DTT pH 8.0). Cells were lysed by addition of lysozyme and sonication and the lysate was cleared by centrifugation. The pellet was washed in inclusion body wash buffer 1 (10 mM Tris-HCl, 10 mM EDTA, 100 mM NaCl, 4 M urea, 2% Triton X-100, pH 8.0) and inclusion body wash buffer 2 (10 mM Tris-HCl, 1 M NaCl, pH 8.0) respectively, after which they were suspended in solubilization buffer (20 mM Tris-HCl, 250 mM NaCl, 10 mM imidazole, 6 M guanidine-HCl, pH 8.0) and stirred at room temperature for 6 hours. Solubilized inclusion bodies were centrifuged at 10000 rpm for 30 minutes at 4°C and supernatants were collected. Supernatants were passed through Ni-NTA agarose beads and washed with 10 column volumes of each wash buffer 1 (20 mM Tris-HCl, 250 mM NaCl, 6 M Gu-HCl, pH 8.0) and wash buffer 2 (20 mM Tris-HCl, 250 mM NaCl, 8 M urea, pH 8.0). Bound protein was eluted with 5 column volumes of 20 mM Tris-HCl, 1 M imidazole, 8 M urea, 20 mM β-mercaptoethanol, pH 8.0. Ni-NTA eluates were concentrated by Centriprep column of 10-kDa cutoff (Millipore) and refolded by rapid dilution technique. For refolding, concentrated protein (2 mg/ml) was added to the refolding buffer (20 mM Tris-HCl, 100 mM NaCl, 2 M urea, 1 mM reduced glutathione, 0.25 mM oxidized glutathione, pH 8.0) in 1∶40 ratio and kept with stirring at 10°C for 48 hours for refolding. Protein was dialyzed against 50 mM Na_2_HPO4, 500 mM NaCl, pH 7.5.

For purification of SERA5 PD, cells were lysed in buffer (50 mM NaH_2_PO_4_, 300 mM NaCl, 10 mM imdazole, pH 8.0) by addition of lysozyme and sonication and the lysate was cleared by centrifugation. Cleared lysate was passed through Ni-NTA agarose and then washed extensively with wash buffer (50 mM NaH_2_PO_4_, 300 mM NaCl, 20 mM imidazole, pH 8.0). Bound protein was eluted by step-wise gradient of imidazole in buffers (50 mM NaH_2_PO_4_, 300 mM NaCl, pH 8.0) and analyzed by SDS-PAGE. Protein was further purified by gel-permeation chromatography. Fractions containing recombinant protein were resolved on 16/60 Superdex 75 gel permeation column (Pharmacia Biotech) in buffer (50 mM Na_2_HPO_4_, 500 mM NaCl, pH 7.5) with a flow rate of 0.5 ml/min and absorbance was measured at 214 nm. Individual peaks were collected and analyzed by SDS-PAGE.

### Synthesis and purification of peptide, DNSDNMF

Peptide, DNSDNMF (D^560^ to F^566^ of SERA5 sequence) was synthesized by solid phase method using Fmoc chemistry at a scale of 0.2 mM, on Rink Amide MHBA resin (Novabiochem). The peptide was purified by reversed phase-HPLC, using a semipreparative C18 column (Phenomenex) on HPLC system (Shimadzu). Solvent gradient was made by solvent A (water with 0.1% TFA) and solvent B (acetonitrile with 0.1% TFA) and the peptide was eluted by a 1% per minute gradient of solvent B from 5% to 75% and monitored by absorbance at 214 nm. Purified peptide was identified by electrospray ionization-mass spectrometry.

### Treatment of *P. falciparum* culture with SERA5 PD

To assess the effect of SERA5 PD on *P. falciparum* growth, parasite culture was synchronized by sorbitol treatment. Synchronous culture at ring stage (6–8 hours) at 2% parasitemia was incubated with SERA5 PD (in PBS, pH 7.4) in 1 µM, 100 nM, 10 nM and 1 nM concentrations (in triplicate) in 96-well flat-bottomed cell culture plate (NUNC) along with cysteine protease inhibitor, E-64 at 10 µM concentration (in triplicate) as positive control. A control protein, *P. falciparum* high mobility group box 2 protein (HMGB2) (in PBS, pH 7.4) was used as a negative control at a concentration of 5 µM (in triplicate). After 48 hours of incubation, percentage of late stage parasites was calculated from the Geimsa-stained smears of infected RBCs by counting nearly 4000 RBCs. To study the effect of SERA5 PD specifically on schizont rupture, parasite culture was treated similarly at late trophozoite stage and Geimsa-stained smears were observed after 24 hours of treatment by counting nearly 4000 RBCs.

### Localization of FITC-labeled SERA5 PD in infected RBCs (iRBCs)

Purified SERA5 PD was incubated with NHS-FITC (Calbiochem) in 10-fold molar excess of amino groups in the protein (20 amino groups) and incubated at room temperature with gentle rocking for 45 minutes. Excess FITC was removed by dialysis in PBS, pH 7.4. Measuring absorbance at 490 nm against buffer blank checked labeling of the protein. Parasite culture at ring stage (2% parasitemia) was incubated with FITC-labeled protein (100 nM) was incubated at a concentration of with the culture in 2% ring stage in 96-well flat-bottomed culture plate for 48 hours. Parasites were counterstained with DAPI and smears of parasites were observed by fluorescence microscopy for FITC and DAPI signals. Besides, parasite pellets from cultures treated with recombinant SERA5 PD (100 nM) and HMGB2 (5 µM) were purified by saponin lysis and lysed in Laemmli sample buffer. Lysate was resolved by SDS-PAGE, transferred on nitrocellulose membrane and probed with horseradish peroxidase anti-hexahistidine antibody. Blot was developed with enhanced chemiluminiscence kit (Pierce).

### Study of interaction of peptide, DNSDNMF with SERA5 C

Interaction of peptide, DNSDNMF with SERA5 C was determined by circular dichroism (CD) spectroscopy and spectrofluorimetry. To study the interaction by CD spectroscopy, CD spectra of SERA5 C in PBS, pH 7.4 (5 µM) alone and with 5 µM peptide were recorded on Jasco spectropolarimeter (model J-810) at 25°C. Scanning speed was 100 nm/min, band width was 1 nm and cell volume was 0.4 ml. To study the interaction of DNSDNMF with SERA5 C by spectrofluorimetry, tryptophan fluorescence emission spectra of SERA5 C in PBS, pH 7.4 (1 µM) alone and with 1 µM peptide were recorded on LS 50B spectrofluorimeter (Perkin Elmer). Parameters for spectrum measurement were; excitation wavelength: 280 nm, emission wavelength range: 290–500 nm, scanning speed: 900 nm/min, excitation slit width: 5 nm, emission slit width: 10 nm, number of accumulations: 5).

### Treatment of *P. falciparum* culture with peptide, DNSDNMF

To assess the effect of peptide DNSDNMF on *P. falciparum* growth, a synchronous ring stage culture at 2% parasitemia was incubated with peptide solution in water in 100 µM and 10 µM concentrations in a 96-well flat-bottomed cell culture plate (NUNC) (in triplicate) along with E-64 in 10 µM concentration (in triplicate). A control peptide, SIINFEKL in 100 µM concentration in water (in triplicate) was taken as negative control. After 48 hours of incubation, percentage of late stage parasites were calculated from the Geimsa-stained smears of infected RBCs by counting nearly 4000 RBCs.

## Supporting Information

Figure S1 **Purification of SERA5 catalytic domain and prodomain.** Panel A represents Ni-NTA purified SERA5 C (marked by an arrow). Panel B represents purification profile of SERA5 PD by size exclusion chromatography. Position of the eluted protein peak is marked by an arrow, which was resolved by SDS-PAGE (inset).
(TIF)

Figure S2 **Purification of synthetic peptide, DNSDNMF.** Synthetic peptide, DNSDNMF was purified on semipreparative RP-HPLC column (Phenomenex). Purified peptide peak is marked by an arrow (Panel A). Identity of the purified peptide was confirmed by electrospray ionization-mass spectrometry. Experimental mass of the peptide peak was 841.3611 Da that was close to the expected mass of 840.85 Da (Panel B).
(TIF)
