# Supplementary figures and images for: Inhibitory Potential of Prodomain of Plasmodium falciparum Protease Serine Repeat Antigen 5 for Asexual Blood Stages of Parasite
Source: PLoS One. 2012 Jan 24;7(1):e30452. doi: 10.1371/journal.pone.0030452 (PMC3265493; doi:10.1371/journal.pone.0030452)

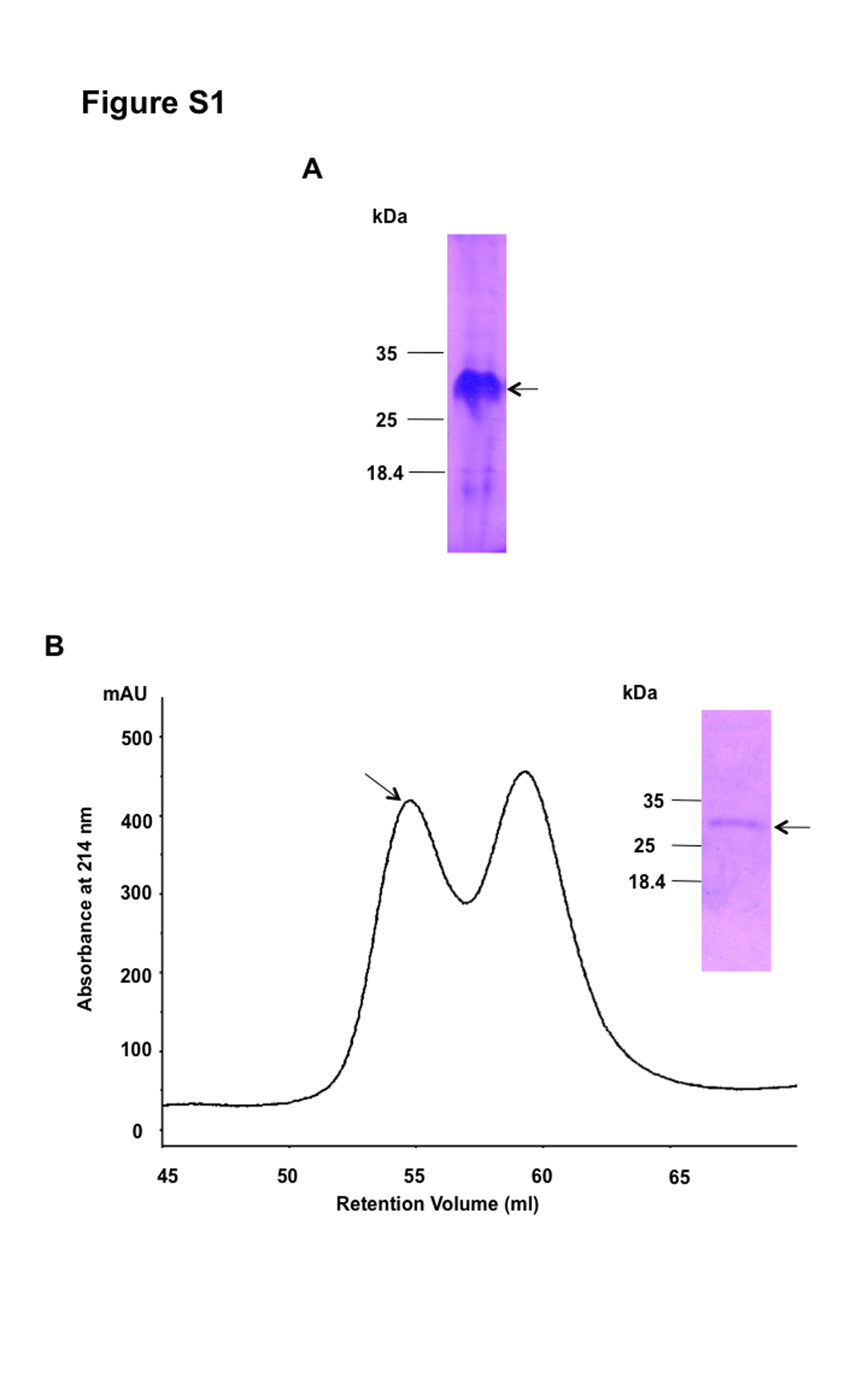

Supplement: Figure S1 — Purification of SERA5 catalytic domain and prodomain. Panel A represents Ni-NTA purified SERA5 C (marked by an arrow). Panel B represents purification profile of SERA5 PD by size exclusion chromatography. Position of the eluted protein peak is marked by an arrow, which was resolved by SDS-PAGE (inset). (TIF) [file pone.0030452.s001.tif]

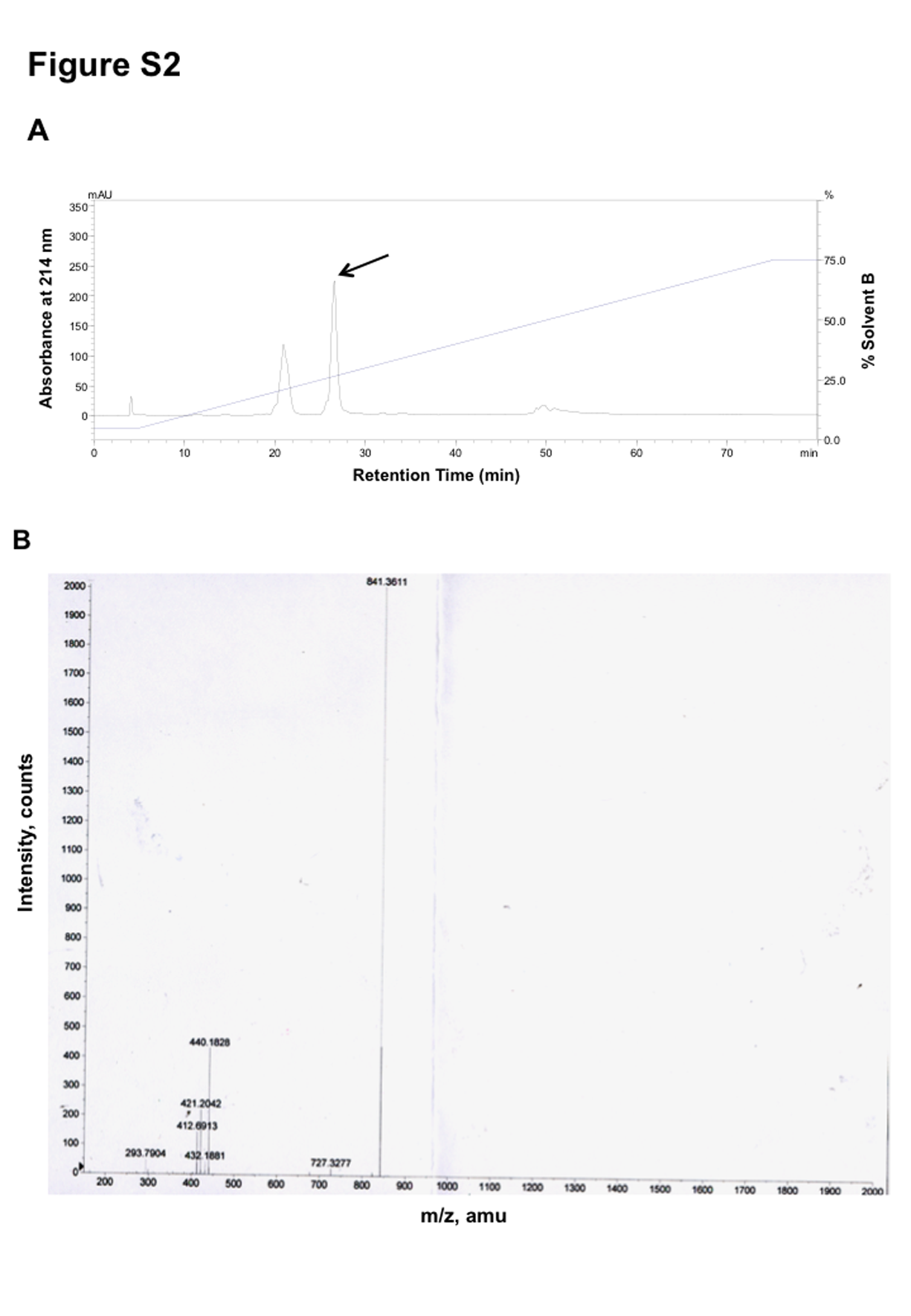

Supplement: Figure S2 — Purification of synthetic peptide, DNSDNMF. Synthetic peptide, DNSDNMF was purified on semipreparative RP-HPLC column (Phenomenex). Purified peptide peak is marked by an arrow (Panel A). Identity of the purified peptide was confirmed by electrospray ionization-mass spectrometry. Experimental mass of the peptide peak was 841.3611 Da that was close to the expected mass of 840.85 Da (Panel B). (TIF) [file pone.0030452.s002.tif]
